# Genetic Variation in an Experimental Goldfish Derived From Hybridization

**DOI:** 10.3389/fgene.2020.595959

**Published:** 2020-12-15

**Authors:** Jing Wang, Weiguo He, Jinfeng Zeng, Lixin Li, Guigui Zhang, Tangluo Li, Caixia Xiang, Mingli Chai, Shaojun Liu

**Affiliations:** ^1^State Key Laboratory of Developmental Biology of Freshwater Fish, College of Life Sciences, Hunan Normal University, Changsha, China; ^2^Department of Histology and Embryology, Clinical Anatomy and Reproductive Medicine Application Institute, Hengyang Medical School, University of South China, Hengyang, China; ^3^Hunan Province Cooperative Innovation Center for Molecular Target New Drug Study Institute of Pharmacy and Pharmacology, University of South China, Hengyang, China

**Keywords:** hybridization, goldfish, genetic variation, speciation, evolution

## Abstract

Owning to the extreme difficulty in identifying the primary generation (G_0_), the common ancestor of various twin-tail goldfish strains remains unclear. However, several authors have hypothesized that this ancestor may have been the crucian carp (*Carassius auratus*). Previously, we generated an experimental hybrid goldfish (EG) from the interspecific hybridization of red crucian carp (*Carassius auratus* ♀, RCC) × common carp (*Cyprinus carpio* ♂, CC). Unlike either parent, EG possessed twin caudal fins similar to those of natural goldfish (*Carassius auratus*, NG). The genetic characteristics of EG, as well as the mechanisms underlying its formation, are largely unknown. Here, we identified the genetic variation in the *chordin* gene that was associated with the formation of the twin-tail phenotype in EG: a stop codon mutation at the 127^th^ amino acid. Furthermore, simple sequence repeat (SSR) genotyping indicated that, among the six alleles, all of the EG alleles were also present in female parent (RCC), but alleles specific to the male parent (CC) were completely lost. At some loci, EG and NG alleles differed, showing that these morphologically similar goldfish were genetically dissimilar. Collectively, our results demonstrated that genetic variations and differentiation contributed to the changes of morphological characteristics in hybrid offspring. This analysis of genetic variation in EG sheds new light on the common ancestor of NG, as well as on the role of hybridization and artificial breeding in NG speciation.

## Introduction

The basic architecture of the vertebrate tail is highly conserved across all vertebrates (Janvier, 1996; Kardong, 2011), including fish, even though fish caudal fins possess divergent and complex skeletal elements (Abe et al., 2014). Interestingly, in certain natural goldfish (*Carassius auratus*, NG) lineages, this conserved architecture has undergone extreme modifications due to artificial selection (Watase, 1887; Bateson, 1894; Matsui, 1934; Chen, 1956; Smartt, 2001). Indeed, some NG strains have twin-tail, which are of considerable ornamental interest (Matsui, 1934; Smartt, 2001). Historical accounts indicate that NG domestication in China began at around 1000 C.E., but the earliest record of twin-tail NG dates from 1596 C.E., suggesting that twin-tail NG emerged approximately 600 years after initial domestication (Chen, 1956; Smartt, 2001). It has been shown that a mutation at the 127^th^ amino-acid position of the *chordin* gene, wherein the glutamic codon is mutated to a stop codon, gives rise to the bifurcated caudal fin (the twin-tail) in NG during ventralization in early embryonic development (Abe et al., 2014). This mutation of the *chordin* gene may have appeared during the domestication period (Abe et al., 2014).

Hybridization, defined as reproduction between members of genetically distinct populations that produces offspring with mixed ancestry, is common in nature and has wide-ranging effects on speciation and population evolution (Barton and Hewitt, 1989; Barton, 2001; Mallet, 2007, 2008; Abbott et al., 2013). Hybridization typically produces heterozygous offspring, which inherit genetic material from both parent genetic material and express biological characteristics intermediate between parents. Numerous species have hybrid ancestry, presumably due to hybridization between closely related species (Cui et al., 2013; Schumer et al., 2016; Meier et al., 2017). In fish, the common carp (CC) and the crucian carp (*Carassius carassius*) belong to the same subfamily, Cyprininae. Previous phylogenic studies have shown that NG are closely related to crucian carp (Komiyama et al., 2009; Gao et al., 2012; Wang et al., 2013). Thus, it has been hypothesized that NG are derived from crucian carp, and may represent a variant of this species (Wheeler, 2000; Smith and McVeagh, 2005). However, as NG is not recently derived, it is extremely difficult or even impossible to reconstruct its exact origin and evolutionary history (James and Abbott, 2005). Thus, we still relatively know little about the precise processes of NG evolution and domestication following hybridization; the exact point at which twin-tail arose, as well as the underlying causes of this phenotype, also remain elusive.

Previously, we obtained an experimental hybrid goldfish (EG) from the hybridization of female red crucian carp (*Carassius carassius*, RCC) × male common carp (*Cyprinus carpio*, CC) after sequential selective breeding. A stable population of EG was successfully established by self-mating (Wang et al., 2014). EG provides an ideal model to study not only the origin and evolution of NG, but also the effects of hybridization on the generation of new species. Consequently, it is important to carefully investigate the genetic characteristics and formation mechanisms of EG.

Here, we identified a stop codon mutation at the 127^th^ amino acid of *chordin* gene, which is associated with the formation of twin-tail, in EG. We also found that the SSR alleles of EG were biased toward the female parent (RCC), and alleles differed between EG and NG at some loci. Collectively, our results showed that certain genetic characteristics of EG, including the *chordin* gene mutation and the RCC-aligned SSR alleles, originated from the distant hybridization, and contributed to the observed difference in morphology. The process of EG generation, as well as the genetic characters of this hybrid, shed new light on the common ancestor of NG, as well as on the role of hybridization and artificial breeding in NG speciation.

## Materials and Methods

### Experimental Fish

All RCC, CC, and EG were raised in ponds at the State Key Laboratory of Developmental Biology of Freshwater Fish, Hunan Normal University, Changsha, China. NG were purchased at a local market. All experiments were approved by the Animal Care Committee of Hunan Normal University and followed the guidelines of the Administration of Affairs Concerning Animal Experimentation of China.

### Cloning and Sequence Analysis of the *Chordin* Gene

To compare the sequences of the *chordin* gene between single-tail and twin-tail fish, we isolated and sequenced the 1^st^ to 6^th^ exons of *chordin* homologues from the embryonic cDNA pools of four fish: single-tail RCC and CC, and twin-tail EG and NG. After self-mating, total RNA was extracted from the gastrula-stage embryos of all four fish using Trizol (Invitrogen, Camarillo, CA, United States). The first-strand cDNA of the *chordin* gene was synthesized using ReverTra Ace (Toyobo, Osaka, Japan). The forward primer 5′-GCGTTACCCATCCAACC-3′ and the reverse primer 5′-TCTGTRTCCGCTTGTGGT-3′ were designed based on CDS of the *chordin* genes from *Carassius auratus* (AB874473.1) and *Cyprinus carpio* (LC092194.1), which were downloaded from GenBank. Each PCR (25 μL) contained 20 ng of cDNA template, 1.5 mM MgCl_2_, 0.2 mM of each dNTP, 0.4 μM of each primer, 1 × PCR buffer, and 1.25 U Taq polymerase (Takara, Dalian, China). The cycling conditions were as follows: an initial denaturation at 94°C for 4 min; followed by 30 cycles of 94°C for 30 s, 55°C for 30 s, and 72°C for 1 min; and a final extension at 72°C for 10 min. PCR products were separated and purified using 1.2% agarose gels and Gel Extraction Kits (Sangon, Shanghai, China), respectively. Purified products were ligated into pMD18-T vectors and transfected into *Escherichia coli* DH5α. Positive clones were sequenced and further analyzed using BLAST^1^ and CLUSTAL W^2^.

### SSR Amplification and Sequencing

We randomly selected eight fish of each type (RCC, CC, NG, and EG) for SSR testing. Total genomic DNA was extracted from the fin tissue of each fish with a DNA extraction kit (Sangon, Shanghai, China). The microsatellite regions were PCR amplified using six florescent-labeled microsatellite primers (synthesized by Sangon, Shanghai, China): three from the common carp (MWF 4, MWF 5, and MWF 16) (Crooijmans et al., 1997); two from the bighead carp (HLJY 3940 and HLJY 2526) (Geng et al., 2006); and one from the crucian carp (MFW1, developed for this study). Primer sequences and annealing temperatures are listed in Supplementary Table 1. PCR amplifications were performed in a total volume of 20 μL, containing 1 μL of genomic DNA (5 ng/μL), 2 μL of 10 × *Taq* Buffer (Mg^2+^ *Plus*), 1 μL of 2.5 mM dNTPs, 0.4 μL of each primer (5 μM), 0.4 μL of *Taq* DNA Polymerase (5 U/μL), and 14.8 μL of ddH_2_O. The PCR cycling conditions were as follows: an initial denaturation at 94°C for 3 min; followed by 35 cycles of 94°C for 30 s, a primer-specific annealing temperature for 30 s, and 72°C for 45 s; a final extension step of 72°C for 7 min. PCR products were sequenced using capillary electrophoresis on an ABI 3730XL DNA sequencer (Applied Biosystems, Foster City, CA, United States) using BigDye Terminator Cycle Sequencing kits (Applied Biosystems, Foster City, CA, United States).

### Genetic Analysis

Genetic distance and genetic polymorphism indexes, including major allele frequency, numbers of genotypes, numbers of alleles, heterozygosity, and gene diversity, were calculated using Popgene32 (Yeh et al., 2000). SSR genotypes, polymorphic information content (PIC) values and the cluster analysis of the 4 populations, which was performed by UPGMA method basing on the genetic distance, were determined with PowerMarker 3.25 (Liu and Muse, 2005).

## Results

### Genetic Variation in the *Chordin* Gene

**FIGURE 1:**
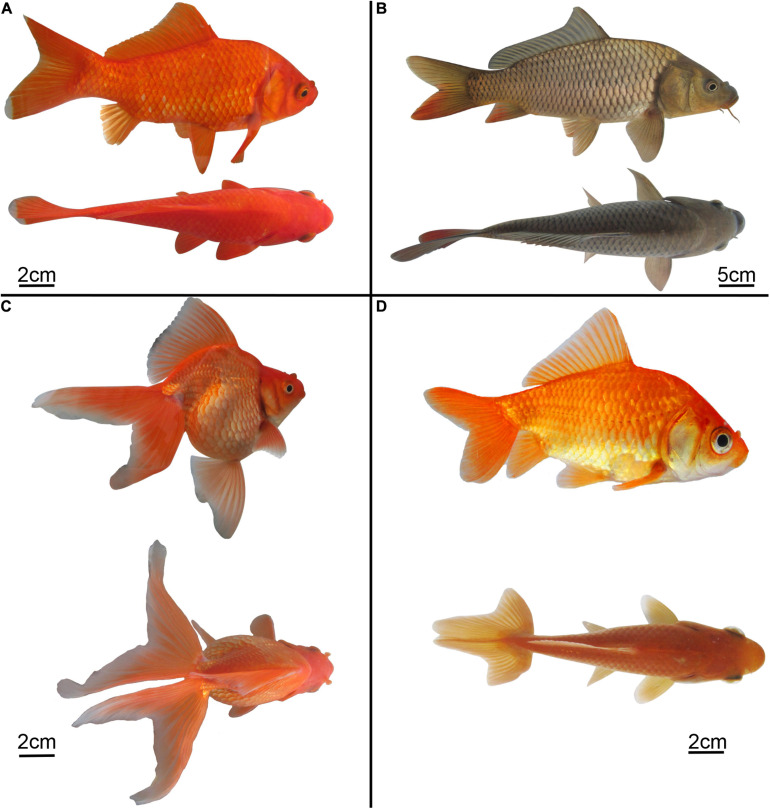
Lateral and dorsal views of phenotypically-representative examples of the four fish taxa included in this study. **(A)** Red crucian carp (RCC, *Carassius auratus*). **(B)** Common carp (CC, *Cyprinus carpio*). **(C)** Natural/wild-type goldfish (NG, *Carassius auratus*). **(D)** Experimental hybrid goldfish (EG). Scale bars, 2 cm **(A,C,D)**, 5 cm **(B)**.

**FIGURE 2:**
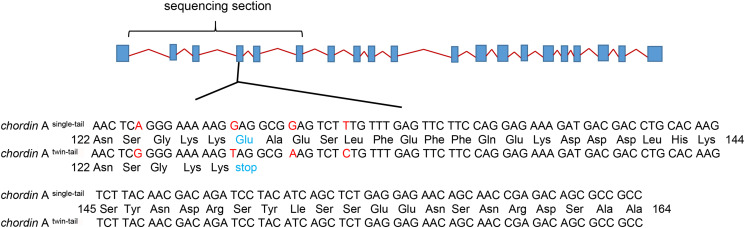
The *chordin* A^*wt*^ and *chordin* A^*E*127X^ amino acid sequences in single-tail and twin-tail fish. Diagram shows the composition of *chordin* A; exons are shown as light green boxes. The twin-tail-specific mutation changes a glutamic acid codon (GAG) to a stop codon (TAG) at amino acid position 127 of the 4^th^ exon; the mutated amino acid is indicated by red letters. The sequenced exons (1^st^ to 6^th^) are located in the horizontal bracket. The lower panel shows the amino acid composition of the 4^th^ exon, which includes the mutation.

Both RCC and CC have single-tail (Figures 1A,B), while EG and NG have twin-tail (Figures 1C,D). To investigate the formation of the twin-tail phenotype in EG, the 1^st^ to 6^th^ exons of *chordin* homologues were cloned from RCC, CC, EG, and NG. All cloned sequences were identified as *chordin* and were submitted to GenBank (RCC, MN918649; CC, MN918650; EG, MN918651; NG, MN918652). There was high nucleotide sequence similarity (> 99%) among the *chordin* genes from all four fish (Supplementary Figure 1). Further analysis showed that *chordin* gene sequences from RCC and CC, the single-tail fish, were identical, as were the *chordin* sequences of EG and NG, the twin-tail fish. However, the *chordin* sequences between the twin-tail and single-tail fish differed: the twin-tail fish had a stop codon (TAG) at the 127^th^ amino acid position of the chordin protein, while the single-tail fish had a glutamic acid codon (GAG) (Figure 2).

### SSR Sequencing and Genotyping

**FIGURE 3:**
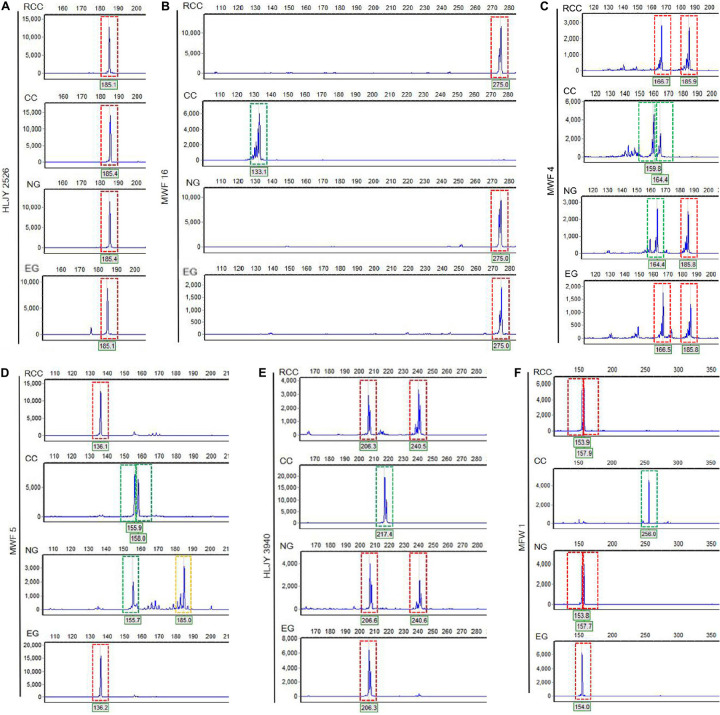
Electropherograms showing allelic peaks at six loci across the four fish lines. **(A)** HLJY 2526 locus. **(B)** MWF 16 locus. **(C)** MWF 4 locus. **(D)** MWF 5 locus. **(E)** HLJY 3940 locus. **(F)** MFW 1 locus. Peaks boxed in red are unique to the crucian carp; peaks boxed in green are unique to the common carp; peaks boxed in yellow are unique to the natural goldfish. The *x*-axes show the size of each segment, and the *y*-axes indicate the strength of the corresponding signal.

**FIGURE 4:**
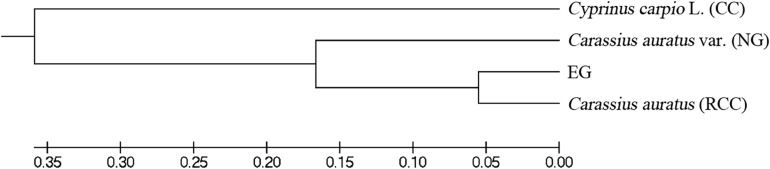
Phylogenetic trees constructed basing on the genetic distance.

Across the four fish (EG, NG, RCC, and CC), each of the six amplified SSR loci (121–302 bp) had 1–8 alleles (Table 1). Almost all the alleles identified in EG, as well as most in NG, were also found in RCC (Table 1). However, nearly all the alleles (except one in HLJY 2526) identified in CC were absent in EG and RCC. The peaks at the HLJY 2526 loci were identical across all four fish (Figure 3A). However, at the MWF 16 locus, RCC, EG, and NG had a peak at 275 bp, while CC had a peak at 133 bp instead (Figure 3B). The peak patterns at the MFW 1 loci presented similar situation as MWF 16 where CC was different from RCC, NG and EG (Figure 3F), and higher similarity to HLJY3940 (Figure 3E). Like RCC and NG, EG had a peak at 206 bp at allele HLJY 3940; however, unlike RCC and NG, EG lacked a peak at 241 bp (Figure 3E). Indeed, all EG alleles also appeared in RCC. In contrast, NG exhibited a specific allele of MWF 5 (at 185 bp) (Figure 3D). In addition, NG respectively presented one allele similar to CC at MWF 4 (at 164 bp) (Figure 3C) and MWF 5 (at 156 bp) (Figure 3D). These results indicated that, although EG and NG appeared morphologically similar, these fish differed genetically. Genetic polymorphism analyses indicated that, of the four populations investigated (RCC, CC, NG, and EG), EG had the lowest polymorphism indexes, corresponding to the highest homogeneity (Table 2). In addition, across all pairs of taxa, genetic distance was lowest between EG and RCC (0.1103; Table 3). Consistent with this, the UPGMA phylogenetic tree recovered EG and RCC as a sister taxa. NG, EG, and RCC formed a single cluster, distinct from CC (Figure 4).

**TABLE 1:** Simple sequence repeat (SSR) genotypes of the four fish lines included in this study: red crucian carp (RCC, *Carassius auratus*); common carp (CC, *Cyprinus carpio*); natural goldfish (NG, *Carassius auratus*); experimental hybrid goldfish (EG).

Locus	RCC	CC	NG	EG
MFW 1	AA/AB/BB	CC/DD	AB/BB	AA
MWF 4	CG/CF	AA/AB/AC/AD	BG	CG
MWF 5	BE/AA	BB/BC	BF/BD	AA/BE
MWF 16	AA	EF/BB/CC/AD/GG/HH	AA	AA
HLJY 3940	AD/AE	BB	AC/AD	AA
HLJY 2526	AA	AA	AA	AA

*Capital letters correspond to allele types at each locus.*

**TABLE 2:** Genetic polymorphism indexes for the four fish lines included in this study: RCC, CC, EG, NG.

	Major allele frequency	Number of genotypes	Number of alleles	Gene diversity	Heterozygosity	Polymorphism information content
RCC	0.7500	1.8333	2.1667	0.33384	0.4048	0.2981
CC	0.7262	3.0000	3.3333	0.3605	0.2143	0.3385
NG	0.7024	1.6667	2.1667	0.3571	0.5476	0.3055
EG	0.8571	1.5000	1.8333	0.1854	0.1667	0.1641

**TABLE 3:** The average genetic distances between each pair of fish taxa included in this study: RCC, CC, EG, NG.

	RCC	CC	NG	EG
RCC	–	0.7281	0.3095	0.1103
CC	0.7281	–	0.6752	0.7492
NG	0.3095	0.6752	–	0.3560
EG	0.1103	0.7492	0.3560	–

## Discussion

Given the uncertainty surrounding the common ancestor of NG strains, as well as the evolutionary processes underlying their emergence (James and Abbott, 2005), the formation and subsequentially stable inheritance of transgressive phenotypes in EG provides a unique opportunity to study the origin and evolution of NG (Wang et al., 2014). That is, studies of the genetic characteristics and formation mechanisms of EG will improve our understanding of the genetic characteristics and formation mechanisms of NG.

### Hybridization and the Formation of Twin-Tail

Hybridization is an important method of animal breeding because this process increases genetic variation (Bullini, 1994), and because hybrids frequently present transgressive segregation exceeding the range between the parental means (Slatkin and Lande, 1994; Rieseberg et al., 1999). The morphological characteristics of EG were highly similar to those of NG, but differed from the original parents RCC and CC: unlike RCC and CC, EG had twin-tail, a spherical body, a short caudal peduncle, and a range of body coloration patterns (Wang et al., 2014). Of these morphological characters, the most recognizable is the twin-tail, as most other fish species have a single tail.

In NG, the twin-tail trait is associated with mutations in the 4^th^ exon of the *chordin* gene (Takashima et al., 2007; Abe et al., 2014). In NG, two alleles of the *chordin* A gene have been identified: the wild-type allele (*chordin* A^*wt*^) includes a glutamic codon at the 127^th^ amino-acid position, while the other allele (*chordin*A^*E*127X^) includes a stop codon at the same position. The allele *chordin*A^*E*127X^ is predicted to encode a truncated protein that contributes to the formation of the twin-tail (Abe et al., 2014). In zebrafish, dysfunction in the *chordin* gene gave rise to a twin-tail phenotype (Schulte-Merker et al., 1997; Fisher and Halpern, 1999). In EG, the potential primary generation (G_0_) of NG, we identified the twin-tail-associated mutation at the 127^th^ amino-acid position in the *chordin* gene: a stop codon (TAG) instead of a glutamic acid codon (GAG). This mutation was identical to that found in NG. In contrast, the *chordin* genes of RCC and CC had a wild-type glutamic acid codon at the same position (Figure 2). These results in the G_0_ generation of EG are consistent with previous studies of twin-tail formation (Abe et al., 2014), and provide direct evidence that this mutation, as well as the associated phenotype (i.e., twin-tail), might have arisen due to hybridization.

### Genetic Bias in EG

SSRs (or microsatellite markers) have been broadly used in studies of molecular evolution (Chistiakov et al., 2006). These simple tandem-repeats are abundantly and randomly distributed throughout genomes, and due to their high polymorphism and rapid evolutionary rate, microsatellite loci are useful for analyses of genetic diversity, population structure, gene flow, and hybridization (Pritchard et al., 2000; Wang et al., 2014). SSR genotyping, which is based on the numbers of repeats, may improve our understanding of genetic relationships within populations or among close relatives (Roy et al., 1994). SSR allele inheritance proceeds in accordance with Mendelian laws of segregation and independent assortment. That is, in diploids, each offspring inherits one copy of each microsatellite segment from each parent (Tesson et al., 2013). Thus, EG, derived from the hybridization of RCC and CC, should have inherited both sets of parental microsatellites. However, among the six alleles, which showed good reproducibility and stability between RCC and CC, we only identified RCC alleles in EG; all alleles unique to CC were completely lost (Table 1 and Figure 3). It may be that the inheritance of SSR alleles by EG did not conform to Mendelian laws because the EG lineage was subject to intense artificial selection. As a result of this selective pressure, the EG population did not contain all the alleles from RCC and CC.

Large-scale morphological changes, which require extensive modifications of developmental mechanisms, are often presumed to require relatively long periods of evolutionary time (Janvier, 2002; Kardong, 2011). However, previous studies have shown that gene duplication and subsequent artificial selection generated dramatic morphological and developmental changes in NG strains within 600 years (Abe et al., 2014). Previously, using a combination of hybridization and artificial selection, we established a stable EG lineage in even less time (F_1_–F_6_, within 6 years) (Wang et al., 2014). Here, our results suggested that the hybridization of female RCC and male CC led to a mutation in the *chordin* gene, which contributed to the expression of the twin-tail phenotype. During hybridization, the separation, recombination, and mutation of genetic materials leads to the formation of new varieties (Schumer et al., 2016). Thus, our results reveal the effects of hybridization on the formation of the twin-tail phenotype in EG, and SSR inheritance patterns, shedding new light on the origin and evolution of NG.

## Data Availability Statement

The original contributions presented in the study are included in the article/Supplementary Materials, further inquiries can be directed to the corresponding author/s.

## Ethics Statement

The animal study was reviewed and approved by the Animal Care Committee of Hunan Normal University and followed the guidelines statement of the Administration of Affairs Concerning Animal Experimentation of China. All samples were raised in natural ponds, all dissections were performed with 100 mg/L MS-222 (Sigma-Aldrich, St Louis, MO, United States), and all efforts were made to minimize suffering.

## Author Contributions

JW and WH conceived the research, analyzed the data, and wrote the manuscript. JZ, LL, GZ, TL, CX, and MC performed the research and writing-reviewed the manuscript. SL provided substantial contributions to conception and coordination. All authors contributed to the article and approved the submitted version.

## Conflict of Interest

The authors declare that the research was conducted in the absence of any commercial or financial relationships that could be construed as a potential conflict of interest.

## Footnotes

1 http://www.ncbi.nlm.nih.gov

2 http://www.ebi.ac.uk/

## Supplementary Material

Supplementary Figure 1 Nucleotide alignment of the sequenced exons (1^st^ to 6^th^) of the *chordin* gene from the four fish lines (RCC, CC, NG, EG). Asterisks (^∗^) indicate bases that differ among lines. The site of termination codon mutation is highlighted in blue.

Supplementary Table 1 Primer pairs and corresponding annealing temperatures for the six SSR loci used in this study.
