# Corrigendum: Genetic Variation in an Experimental Goldfish Derived From Hybridization

**DOI:** 10.3389/fgene.2021.645346

**Published:** 2021-05-05

**Authors:** Jing Wang, Weiguo He, Jinfeng Zeng, Lixin Li, Guigui Zhang, Tangluo Li, Caixia Xiang, Mingli Chai, Shaojun Liu

**Affiliations:** ^1^State Key Laboratory of Developmental Biology of Freshwater Fish, College of Life Sciences, Hunan Normal University, Changsha, China; ^2^Department of Histology and Embryology, Clinical Anatomy and Reproductive Medicine Application Institute, Hengyang Medical School, University of South China, Hengyang, China; ^3^Hunan Province Cooperative Innovation Center for Molecular Target New Drug Study Institute of Pharmacy and Pharmacology, University of South China, Hengyang, China

**Keywords:** hybridization, goldfish, genetic variation, speciation, evolution

In the original article, there was a mistake in Figure 3 as published. “NG” was erroneously used in both Figures 3A and **B**, where the lower “NG” should actually be “EG.” The corrected Figure 3 appears below.

**Figure 3:**
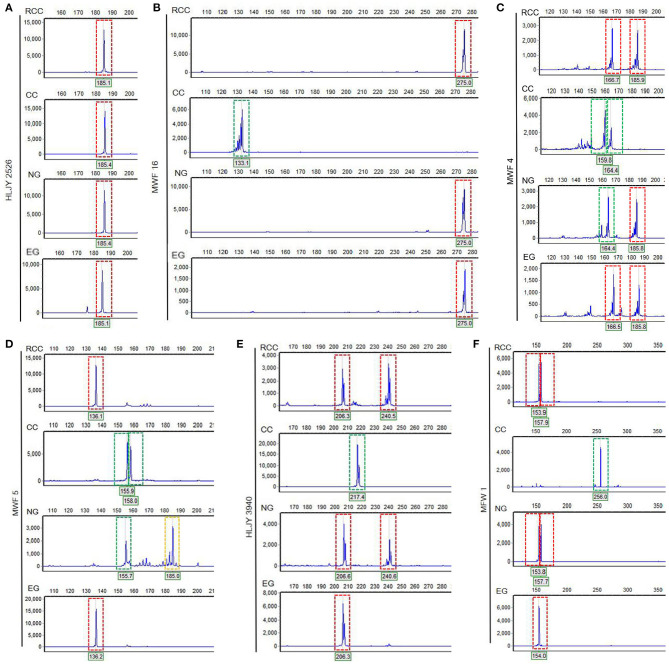
Electropherograms showing allelic peaks at six loci across the four fish lines. **(A)** HLJY 2526 locus. **(B)** MWF 16 locus. **(C)** MWF 4 locus. **(D)** MWF 5 locus. **(E)** HLJY 3940 locus. **(F)** MFW 1 locus. Peaks boxed in red are unique to the crucian carp; peaks boxed in green are unique to the common carp; peaks boxed in yellow are unique to the natural goldfish. The *x*-axes show the size of each segment, and the *y*-axes indicate the strength of the corresponding signal.

Additionally, there was also a mistake in Table 1 as published. The locus names were erroneously written as “MFW 4,” “MFW 5,” and “MFW16” and should actually be “MWF 4,” “MWF 5,” and “MWF 16,” respectively. The corrected Table 1 appears below.

**Table 1:** Simple sequence repeat (SSR) genotypes of the four fish lines included in this study: red crucian carp (RCC, *Carassius auratus*); common carp (CC, *Cyprinus carpio*); natural goldfish (NG, *Carassius auratus*); experimental hybrid goldfish (EG).

**Locus**	**RCC**	**CC**	**NG**	**EG**
MFW 1	AA/AB/BB	CC/DD	AB/BB	AA
MWF 4	CG/CF	AA/AB/AC/AD	BG	CG
MWF 5	BE/AA	BB/BC	BF/BD	AA/BE
MWF 16	AA	EF/BB/CC/AD/GG/HH	AA	AA
HLJY 3940	AD/AE	BB	AC/AD	AA
HLJY 2526	AA	AA	AA	AA

*Capital letters correspond to allele types at each locus*.

Finally, there was also a mistake in the text of the published article. The name of locus “MWF 1” in “The peak patterns at the MWF 1 loci were similar (Figure 3F)” should be “MFW1” and the presentation of this sentence was not clear enough to show the meaning that the peak patterns at the MFW 1 loci were similar to the previous MWF 16 loci, which means that CC is different from the three other lines (Figure 3F). In addition, MFW1 loci presented higher similarity to HLJY3940 (Figure 3E).

There was also an additional error. The sentence “In contrast, NG exhibited a specific allele of MWF 5 (at 185 bp) and was more similar to CC at alleles MWF 4 and MWF 5 (Figures 3C,D)” was also not clear enough.

Corrections have therefore been made to the **Results** section, subsection **SSR Sequencing and Genotyping**:

“Across the four fish (EG, NG, RCC, and CC), each of the six amplified SSR loci (121–302 bp) had 1–8 alleles (Table 1). Almost all the alleles identified in EG, as well as most in NG, were also found in RCC (Table 1). However, nearly all the alleles (except one in HLJY 2526) identified in CC were absent in EG and RCC. The peaks at the HLJY 2526 loci were identical across all four fish (Figure 3A). However, at the MWF 16 locus, RCC, EG, and NG had a peak at 275 bp, while CC had a peak at 133 bp instead (Figure 3B). The peak patterns at the MFW 1 loci presented similar situation as MWF 16 where CC was different from RCC, NG and EG (Figure 3F), and higher similarity to HLJY3940 (Figure 3E). Like RCC and NG, EG had a peak at 206 bp at allele HLJY 3940; however, unlike RCC and NG, EG lacked a peak at 241 bp (Figure 3E). Indeed, all EG alleles also appeared in RCC. In contrast, NG exhibited a specific allele of MWF 5 (at 185 bp) (Figure 3D). In addition, NG respectively presented one allele similar to CC at MWF 4 (at 164 bp) (Figure 3C) and MWF 5 (at 156 bp) (Figure 3D). These results indicated that, although EG and NG appeared morphologically similar, these fish differed genetically. Genetic polymorphism analyses indicated that, of the four populations investigated (RCC, CC, NG, and EG), EG had the lowest polymorphism indexes, corresponding to the highest homogeneity (**Table 2**). In addition, across all pairs of taxa, genetic distance was lowest between EG and RCC (0.1103; **Table 3**). Consistent with this, the UPGMA phylogenetic tree recovered EG and RCC as a sister taxa. NG, EG, and RCC formed a single cluster, distinct from CC (**Figure 4**).”

The authors apologize for these errors and state that this does not change the scientific conclusions of the article in any way. The original article has been updated.

