# Supplementary figures and images for: Genetic Variation in an Experimental Goldfish Derived From Hybridization
Source: Front Genet. 2020 Dec 15;11:595959. doi: 10.3389/fgene.2020.595959 (PMC7770164; doi:10.3389/fgene.2020.595959)

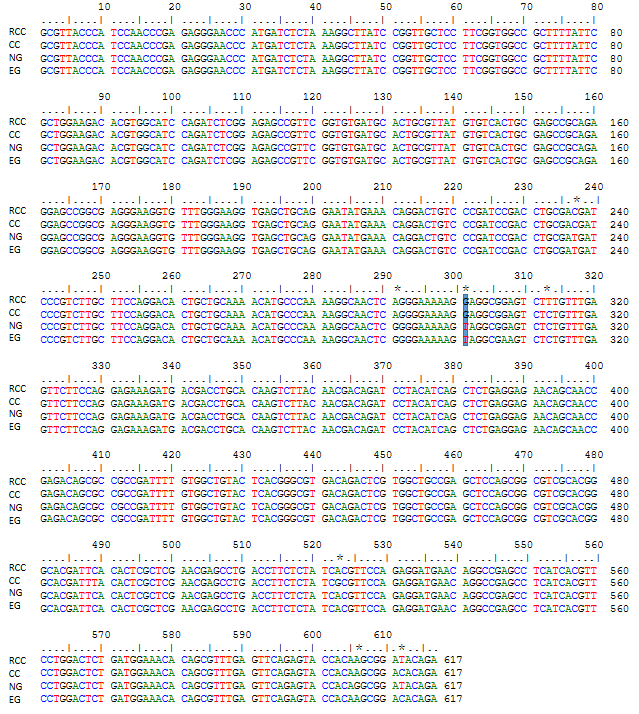

Supplement: Supplementary Figure 1 — Nucleotide alignment of the sequenced exons (1st to 6th) of the chordin gene from the four fish lines (RCC, CC, NG, EG). Asterisks (∗) indicate bases that differ among lines. The site of termination codon mutation is highlighted in blue. [file Image_1.TIF]
