# Supplementary material for: Genetic Variation in an Experimental Goldfish Derived From Hybridization
Source: Front Genet. 2020 Dec 15;11:595959. doi: 10.3389/fgene.2020.595959 (PMC7770164; doi:10.3389/fgene.2020.595959)
Supplement: Supplementary Table 1 — Primer pairs and corresponding annealing temperatures for the six SSR loci used in this study. [file Table_1.docx]

**Appended table 1** Primer pairs and corresponding annealing temperatures for the six SSR loci used in this study.

|  | Primer sequence 5’-3’ | Annealing temperature (°C) |
| --- | --- | --- |
| MWF 4 | F-TCCAAGTCAGTTTAATCACCG | 60 |
|  | R-GGGAAGCGTTGACAACAAGC |  |
| MWF 5 | F-GAGATGCCTGGGGAAGTCAC | 64 |
|  | R-AAAGAGAGCGGGGTAAAGGAG |  |
| MWF 16 | F-GTCCATTGTGTCAAGATAGAG | 64 |
|  | R-TCTTCATTTCAGGCTGCAAAG |  |
| HLJY 3940 | F-GAACGCTCTACGGAATGG | 52 |
|  | R- TCCTGTTACACTATCTGGGT |  |
| HLJY 2526 | F-AACAGCCACATAACCAAT | 50 |
|  | R-AGATAGCCGTTGTCATTC |  |
| MFW 1 | F-AGCGGAACTCACTAAAC | 50 |
|  | R-ACAGGCTTCCAGTAAAA |  |
